# A FAST-BRISK Feature Detector with Depth Information

**DOI:** 10.3390/s18113908

**Published:** 2018-11-13

**Authors:** Yanli Liu, Heng Zhang, Hanlei Guo, Neal N. Xiong

**Affiliations:** 1 School of Information Engineering, East China Jiaotong University, Nanchang 330013, China; hbliuyanli@126.com (Y.L.); guohanlei@126.com (H.G.); 2 Department of Mathematics and Computer Science, Northeastern State University, Tahlequah, OK 74464, USA; xiong31@nsuok.edu

**Keywords:** BRISK (Binary Robust Invariant Scalable Keypoints), depth information, scale factor, scale invariance, rotation invariance

## Abstract

RGB-D cameras offer both color and depth images of the surrounding environment, making them an attractive option for robotic and vision applications. This work introduces the BRISK_D algorithm, which efficiently combines Features from Accelerated Segment Test (FAST) and Binary Robust Invariant Scalable Keypoints (BRISK) methods. In the BRISK_D algorithm, the keypoints are detected by the FAST algorithm and the location of the keypoint is refined in the scale and the space. The scale factor of the keypoint is directly computed with the depth information of the image. In the experiment, we have made a detailed comparative analysis of the three algorithms SURF, BRISK and BRISK_D from the aspects of scaling, rotation, perspective and blur. The BRISK_D algorithm combines depth information and has good algorithm performance.

## 1. Introduction

In the field of machine vision and robotics research, the feature detection has attracted the attention of scholars at home and abroad. This research focuses on the robustness and the invariance to image noise, scale, translation and rotation transformations. Many feature detector methods are available in many fields [1,2,3,4], such as robot navigation, pattern recognition, image and video detection, target tracking, scene classification, texture recognition.

In recent years, many new feature description algorithms have been proposed under the premise of satisfying the invariance of rotation, scale transformation and noise, such as Scale-invariant Feature Transform (SIFT) [5,6], Speeded Up Robust Feature (SURF) [7,8], Binary Robust Independent Elementary Features (BRIEF) [9,10], and Binary Robust Invariant Scalable Keypoints (BRISK) [11,12]. The BRISK algorithm is a feature point detection and description algorithm with scale invariance and rotation invariance. It constructs the feature descriptor of the local image through the gray scale relationship of random point pairs in the neighborhood of the local image, and obtains the binary feature descriptor. Compared with the traditional algorithm, the matching speed of BRISK is faster and the storage memory is lower, but the robustness of BRISK is reduced.

In recent years, the RGB-D sensors represented by Kinect of Microsoft are spreading quickly as the RGB-D sensor can obtain the RGB image and depth image simultaneously. Compared to stereo cameras and Time-Of-Flight cameras, it has many advantages such as low price, information integrity and complex environmental adaptation. So, the RGB-D SLAM based on RGB-D images has quickly become a research focus. But algorithms only based on texture information of 2D image are widely used, such as the SURF algorithm, SIFT algorithm, BRIEF algorithm and BRISK algorithm. These don’t take the depth information of the RGB-D image into account. In this paper, the BRISK algorithm will be improved using the depth information of the RGB-D image and intensity centroid.

In this paper, the keypoints firstly are detected by the Features from Accelerated Segment Test (FAST) algorithm. Then, the location of the keypoint is refined in the scale and the space. Next, the scale factor of the keypoint is directly computed with the depth information of the image. And next, the intensity centroid of the circle centered on the keypoint is calculated, and the orientation of keypoint is computed by the offset from its intensity centroid. Finally, the experimental results show that, compared with the original BRISK algorithm, the improved BRISK algorithm’s robustness of rotation and scale invariance are stronger.

This paper is organized as follows. In Section 2 a brief survey of related work is presented. Then the BRISK algorithm principle is described in Section 3. In Section 4, improvement of the BRISK algorithm (BRISK_D algorithm) is introduced. Some experimental results are presented in Section 5 and, finally, we provide some conclusions and future work.

## 2. Related Work

Attneave et al. [13] explains some of the problems of visual perception. From the perspective of visual behavior, people introduce information theory to quantify the data received by the visual system. From an information point of view, most of the visual information received by the human visual system is redundant, because the information received by the intricate neurons is interrelated. People can extract the main signal content by extracting information from a limited number of neurons. According to the experimental research, it is proposed to express the main visual information by pixels with large degree of variation in the uneven region of the image. However, this theory is only experimentally described, and has not been quantified.

The history of image local feature research first originates from Moravec’s point of interest [14]. It detects interest points based on the similarity of neighborhood pixel autocorrelation functions. It is a relatively original corner detection method, which is particularly sensitive to the pixel gray noise and does not have the feature of the rotation invariance.

The Hessian detector proposed by Beaudet [15] opens the precedent of feature point detection algorithm based on image local gradient information. The Hessian matrix is very suitable for measuring the local gradient features of images. Many well-known feature point detection algorithms based on image gradient information use Hessian matrix to calculate their corresponding feature point response functions, such as LoG [16], SURF [7], KAZE [17], AKAZE [18],and so on.

The first corner detection algorithm is the Harris corner detection method [19]. It uses a differential operator to construct a 2×2 gradient matrix containing structural features and uses the distribution of gradient matrix eigenvalues to determine whether the local structure of the image is a corner, edge or flat region. The Shi-Tomas [20] corner defines the corner point from the discriminability of the feature point tracking. It is essentially the same as the Harris corner, and is a development of the Harris corner. The above corner points do not have scale invariance, nor rotation invariance.

Lindeberg [21,22] introduces multiscale analysis in image processing, and points out that the Gauss kernel filter is the optimal scale filter. Mikolajczyk combines the multi-scale analysis and affine transformation of Lindeberg with the Harris feature points based on the gradient feature to develop the invariant features of Harris-Laplace and Harris-Affine [23]. But the computational complexity is too large and the application is limited.

In addition to gradient-based feature points, the local invariant features appeared in 1999~2006 are more based on the patch feature. Most of the patch characteristics can provide better location, scale and direction information. Among them, the most famous feature is the SIFT feature proposed by D.G. Lowe [5]. Although SIFT is a popular feature detection and descriptor, it has a large amount of computation and storage. SURF [7] is another plaque-based invariant feature. In the integral operation of SURF, Bay draws on Viola’s integral graph technique in face detection [24]. The computing speed is several times faster than that of SIFT, but its matching accuracy is somewhat lower than that of SIFT.

Bay et al. propose the Fast-Hessian feature detector based on the Hessian matrix in the SURF (Speeded Up Robust Features) [25] algorithm and use the integral image to calculate its corresponding descriptor. Compared with the SIFT algorithm, the computational efficiency can be greatly improved. Similarly, Alcantarilla et al. also uses the Hessian matrix for feature point detection, combined with the corresponding descriptors to form KAZE [17] and AKAZE [18] feature detection algorithms.

Since 2006, local invariant feature detectors and descriptors based on binarization features have become the new mainstream in the field of invariant features. The originator of the detector is FAST [26,27], which belongs to the SUSAN [28] operator. The SUSAN operator originates in the literature [29]. Although the FAST operator is computationally efficient, it depends on the scene, and has no direction and scale information. It does not have scale invariance and rotation invariance. In order to overcome the dependence of FAST detector on scene, a new scene independent detector is proposed, which is the Adaptive and Generic Accelerated Segment Test (AGAST) [30] detector. An improvement on FAST is Oriented-FAST, which is a feature detection sub module in the ORB [31] feature generation method. The feature detection part of the BRISK [11] method is based on AGAST, which combines the advantages of SIFT on scale space search and obtains a binary invariant feature detector with scale invariance.

For the binarized feature descriptor, the most famous one is the BRIEF [9,32]. The binarized description feature consists of bit strings, each bit representing a gray-scale contrast between a set of pairs of points distributed at a particular position in the local neighborhood image of the feature point, all of which are used for comparison. It constitutes the constellation of the descriptor. Different feature descriptors use different constellation structures, and the pairs of points selected in the constellation for grayscale contrast to generate binarized features are also different. Since the feature is composed of two points of gray scale comparison, the feature description speed is faster, and the Euclidean distance or the Mahalanobis distance is not used in the feature matching process, and the Hamming Distance is adopted. Hamming distance can be calculated using efficient XOR operation instructions, which greatly improves the speed of feature matching. Therefore, the description and matching based on the binarized feature has higher computational efficiency than the traditional multidimensional real number feature. The improved Steered BRIEF [9] method based on the BRIEF provides a feature orientation that is not available in the BRIEF, making the Steered BRIEF a rotation-invariant feature.

Because in the design of BRIEF, the point-to-point distribution selected in the constellation for comparison does not have a rigorous and scientific design, the binarization features have greater correlation. rBRIEF overcomes this weakness by focusing on selecting the less-relevant binary features to construct the constellation structure of the descriptor. ORB [31] is a feature method that combines Oriented-FAST and rBRIEF. In order to avoid the strict alignment phenomenon required by the similar features and to suppress the influence of gray noise on the feature description, like DAISY [33], BRISK also adds a gray-scale smoothing step before the point-to-scale is compared on the constellation.

The above feature description methods are divided into two categories according to the computational complexity and the storage space size: the first type is a type with large computational complexity and large storage consumption, such as SIFT, SURF, DAISY, and so on, wherein DAISY is suitable for dense matching. Due to the high-dimensional real number vector for feature description, such features are complicated to calculate and difficult to save with large amount of computation.

The second type is a binarization feature with better comprehensive performance, which has less computational complexity and requires less storage space, such as BRIEF, BRISK, ORB, and so on. They both use a binarized string feature to describe the local texture and then use the Hamming distance to describe the difference between the features. They have matching performance comparable to the first type of features, and the computational speed is significantly better than the first type of features, where BRIEF is the prototype of the second type. It has some defects, such as the need for precise alignment, and so on. ORB does not have the scale feature, but the BRISK has better comprehensive performance and has faster calculation speed than the SIFT and SURF.

## 3. BRISK Algorithm Principle

The BRISK algorithm includes three main modules: keypoint detection, keypoint description and descriptor matching. First, the scale space pyramid is constructed, and the stable extreme points of sub-pixel precision in continuous scale space are extracted by AGAST [30] (the Adaptive corner detection operator). Then, the binary feature descriptor of the local image is established by using the gray scale relationship of the random sample point pairs in the local image neighborhood. Finally, the Hamming distance is used for the feature matching.

### 3.1. Scale-Space KeyPoint Detection

**Figure 1:**
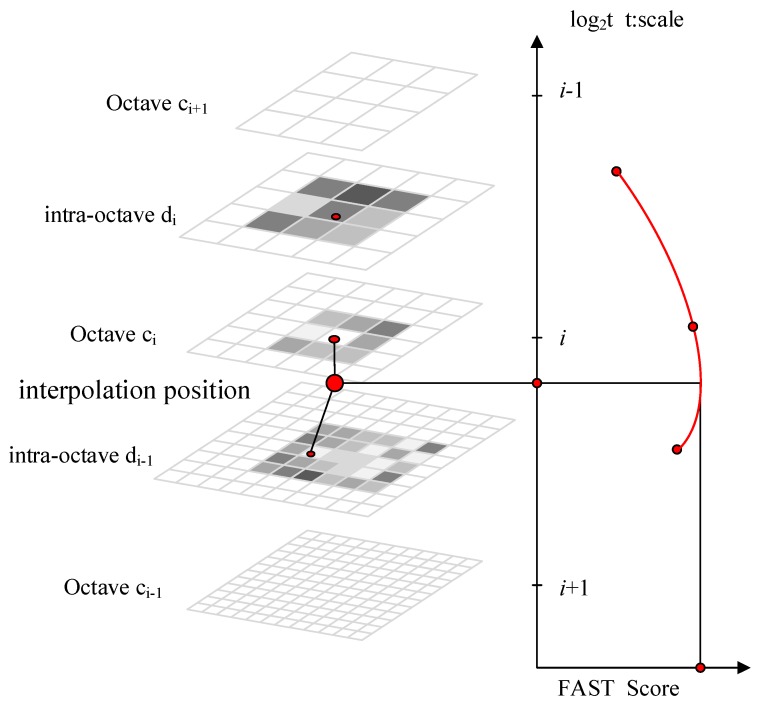
Scale-space interest point detection.

The keypoint detection methodology of BRISK is inspired by AGAST (Adaptive and Generic Accelerated Segment Test) [30]. The FAST (Features from Accelerated Segment Test) [27] is extended to the image plane and the scale-space. In the BRISK algorithm framework, the scale Pyramid space is composed of n octaves $c_{i}$ and n intra-octaves $d_{i}$, where i={0,1,⋯,n−1} and typically n=4. The octaves are formed by progressively half-sampling the original image (corresponding to $C_{0}$). Each intra-octave $d_{i}$ is located between layers $c_{i}$ and $c_{i+1}$(as illustrated in Figure 1). The first intra-octave $d_{0}$ is obtained by down-sampling the original image $C_{0}$ by a factor of 1.5, while the rest of the intra-octave layers are derived by successive half sampling. Therefore, if t denotes scale the $t(c_{i})=2^{i}$ and $t(d_{i})=1.5(2^{i})$.

The keypoint detection algorithm consists of the following two steps. First, the FAST 9-16 detector is applied on each octave and intra-octave separately using the same threshold T to identify potential regions of interest. Next, the points belonging to these regions are subjected to a non-maxima suppression in scale-space. Keypoints must satisfy the following two conditions: (1) the FAST score sc of a point to be detected located in the same layer must be greater than the other eight points adjacent to it; and (2) the scores in the layer above and below will need to be lower the FAST score s of this point. The detection of maxima across the scale axis at octave $c_{0}$ is a special case. In order to obtain the FAST scores for a virtual intra-octave $d_{-1}$ below $c_{0}$, we apply the FAST 5–8 [34] mask on $c_{0}$. However, the scores in patch of $d_{-1}$ are in this case not required to be lower than the score of the examined point in octave $c_{0}$.

Considering image saliency as a continuous quantity not only across the image but also along the scale dimension, we perform a sub-pixel and continuous scale refinement for each detected maximum. In order to limit complexity of the refinement process, we first fit a 2D quadratic function in the least-squares sense to each of the three scores-patches (as obtained in the layer of the keypoint, the one above, and the one below) resulting in three sub-pixel maximal value. In order to avoid resampling, we consider a 3 by 3 score patch on each layer. Next, these refined scores are used to fit a 1D parabola along the scale axis yielding the final score estimate and scale estimate at its maximum. On the final step, we re-interpolate the image coordinates between the patches in the layers.

### 3.2. Keypoint Description

The description of the keypoint has a significant impact on subsequent efficiency of descriptor matching, also influencing the whole performance of the algorithm. Each keypoint of SIFT has a 128-vector descriptor, each keypoint of SURF has a 64-vector descriptor. In the descriptor matching stage, SIFT and SURF can only be matched using Euclidean distance, what is inefficient. Different from SIFT and SURF, BRISK descriptor is descripted by the binary bitstring [30], which is put forward by Michael Calondor and matched by Hamming Distance. In other words, Hamming Distance can be computed very efficiently with a bitwise XOR operation.

**Figure 2:**
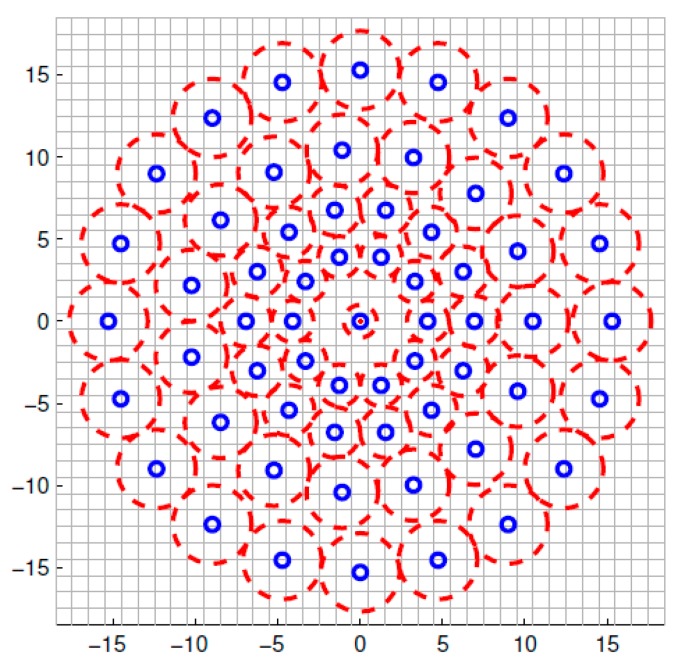
Binary Robust Invariant Scalable Keypoints (BRISK) sampling pattern.

Different from other binary feature description algorithm (such as BRIEF) using a randomly selected point pair, the BRISK descriptor adopts fixed neighborhood sampling pattern to describe feature points. Four concentric circles are built within the block whose size is 40×40 pixels centered on the interest point, and N(N=60) points with uniform distribution and the same spacing are respectively obtained on the four concentric circles. As shown in Figure 2, the small blue circles denote the sampling locations. In order to avoid aliasing effects when sampling the image intensity of a point $p_{i}$ in the pattern, Gaussian smoothing with standard deviation $\sigma_{i}$ proportional to the distance between the points on the respective circle is applied.

Define point pair set formed by all pairs of sample points as A:

(1) $$A=\{(p_{i},p_{j})\in R^{2}\times R^{2}\;|\;i<N\wedge j<i\wedge i,j\in N\}$$

$\left(p_{i},p_{j}\right)$ is point pair of set A.

The gray values smoothed by pixels $p_{i}$ and $p_{j}$ are respectively denoted as $I(p_{i},\sigma_{i})$ and $I(p_{j},\sigma_{j})$. The local gradient between the two pixel points is as follows.

(2) $$g(p_{i},p_{j})=(p_{i}-p_{j})•\frac{I(p_{j},\sigma_{j})-I(p_{i},\sigma_{i})}{∥p_{j}-p_{i}∥}$$

where 1≤i≤N, 1≤j≤N.

According to the distance between pixel pairs, the set of short distance sampling points is defined as S, and the set of long-distance sampling points is defined as L.

(3) $$S=\{(P_{i},P_{j})\in A|\;∥P_{i}-P_{j}∥<\sigma_{\max}\}\subseteq A$$

(4) $$L=\{(P_{i},P_{j})\in A|\;∥P_{i}-P_{j}∥>\sigma_{\min}\}\subseteq A$$

where $\sigma_{\max}$ is long distances threshold, typically $\sigma_{\max}$ is 9.75t, $\sigma_{\min}$ is shot distances threshold, typically $\sigma_{\min}$ is 13.67t, t is the spatial scale of feature points.

In the BRISK algorithm, local gradient is assumed be annihilated each other and the local gradient does not need to be considered in the calculation of the overall gradient mode. Therefore, the overall mode direction of the feature points can be estimated by the set L:

(5) $$g=\left[\begin{array}{l} g_{x}\\ g_{y} \end{array}\right]=\frac{1}{l}•\sum_{(p_{i},p_{j})\in L}g(p_{i},p_{j})$$

where l is the length of subset of long-distance pairings L. $g(p_{i},p_{j})$ denotes the gradient of the feature point pair $\left(p_{i},p_{j}\right)$. $g_{x}$ and $g_{y}$ are gradients sum of the long-distance point pair set on x axis and y axis direction.

In order to build a descriptor with rotation invariance and scale invariance, sampling pattern rotates θ angle around the feature point k. θ is computed by:

(6) $$\theta =\mathrm{actan}2(g_{y},g_{x})$$

Then, gray intensity of short-distance pairs set S is compared and cascaded, feature descriptor is generated according to the formula (7):

(7) $$b=\left\{ \begin{array}{l} \begin{array}{cc} 1 & I(p_{j}^{\theta },\sigma_{j})>I(p_{i}^{\theta },\sigma_{i}) \end{array}\\ \begin{array}{cc} 0 & otherwise \end{array} \end{array}\right.\begin{array}{c} \;(P_{i}^{\theta },P_{j}^{\theta })\in S \end{array}$$

where $p_{i}^{\theta }$ is the point that $p_{i}$ revolves around the feature point k by rotating θ angle. $I(p_{i}^{\theta },\sigma_{i})$ is gray intensity of $I(p_{i}^{\theta },\sigma_{i})$ after rotating θ angle around the feature point k.

### 3.3. BRISK Descriptor Matching

The matching of the descriptors is achieved by comparing the similarities between the descriptors of the two feature points. Because the BRISK algorithm uses the binary bit string composed of 1 and 0 to describe the extracted feature points, the similarity of the descriptors is described by calculating the Hamming distance of the descriptor. The Hamming distance calculation is implemented using a bitwise XOR operation, that is, two values participating in the operation. If their corresponding bits are the same, the result is “0”, otherwise it is “1”. Then, the statistics of “1” are counted and the more the number of “1”, the more dissimilarity of the two descriptors, otherwise the opposite. Assuming X and Y are two BRISK descriptors, then:

(8) $$X=\chi_{1}\chi_{2}\cdots \chi_{i}\cdots \chi_{N}$$

(9) $$Y=\gamma_{1}\gamma_{2}\cdots \gamma_{i}\cdots \gamma_{N}$$

where the value of $x_{i}$ and $y_{i}$ is “1” or “0”.

The Hamming distance equation is given by Equation (10).

(10) $$HD(X,Y)=\sum_{i=1}^{N}\chi_{i}⊕\gamma_{i}=\sum_{i=1}^{n}b(\chi_{i},\gamma_{i})$$

where $b(\chi_{i},\gamma_{i})$ denotes bit inequality, in Equation (10), $\chi_{i}$ and $\gamma_{i}$ are the i-th bits of the descriptors X and Y respectively.

(11) $$b(x,y)=\left\{ \begin{array}{l} 1\;x\neq y\\ 0\;x=y \end{array}\right.$$

The symbol ⊕ is the XOR symbol. The value of Hamming distance is computed to estimate the degree of two BRISK descriptors matching. The greater the value of Hamming distance, the lower the degree of descriptors matching.

## 4. Improvement of BRISK Algorithm (BRISK_D Algorithm)

### 4.1. Improvement Ideas

From the brief analysis above, it can be seen that the BRISK algorithm realizes the scale invariance of descriptors by detecting feature points in multi-scale layer and realizes the rotational invariance of descriptors by determining direction of master mode using long-distance pixel pairs. But after comparing the BRISK algorithm with the SIFT algorithm and the SURF algorithm, robustness of the BRISK algorithm is feeble in aspects of scale invariance and rotational invariance. For that reason, this paper combines the depth information of pixels in RGB-D images to compute the scale factors of descriptors and adopts Intensity Centroid [35] to determine the main directions of descriptors in order to enhance the robustness of descriptor’s scale invariance and rotational invariance.

The BRISK_D algorithm is also divided into three modules: feature point detection, descriptor construction and feature matching. Feature matching is same as it is in the BRISK algorithm.

For the feature point detection: first of all, the threshold value is adjusted to produce appropriate interest points. Then, the location of interest points in scale and space is refined. Assuming the initial position of the interest point in the scale pyramid is (x,y,σ), by finding the refinement term Δx, Δy and Δσ, position of interest point is $\left(\bar{x},\bar{y},\bar{\sigma })=(x+\Delta x,y+\Delta y,\sigma \Delta \sigma \right)$.

Descriptor construction: first of all, with the detected feature points as the center, the pixel pair is selected by the fixed field mode. Then, scale factor is calculated according to the depth information of pixels, and next the main direction of pixels is determined. Finally, the feature points are described according to the gray values of pixels.

**Figure 3:**
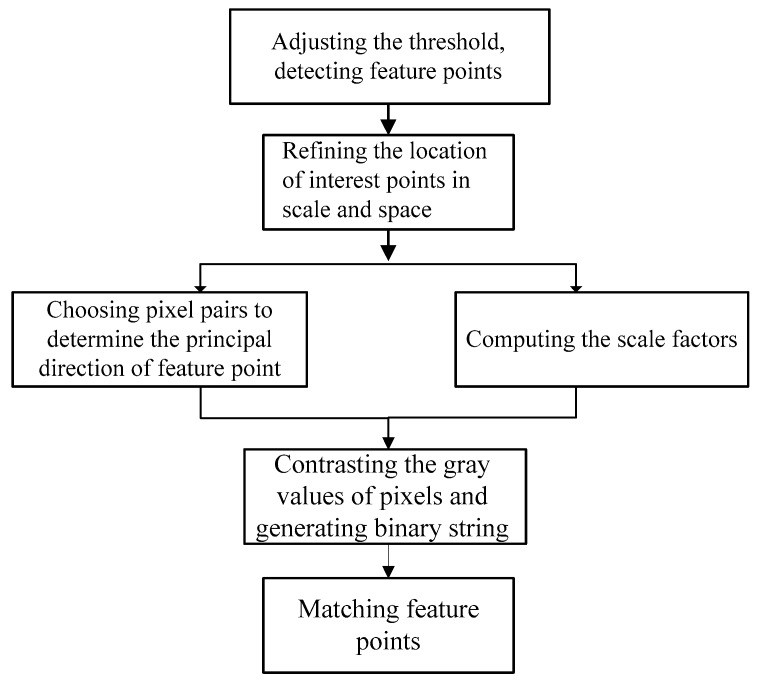
The flow of improved BRISK algorithm.

The algorithm flow is shown in Figure 3.

### 4.2. Precise Location of Interest Points

**Figure 4:**
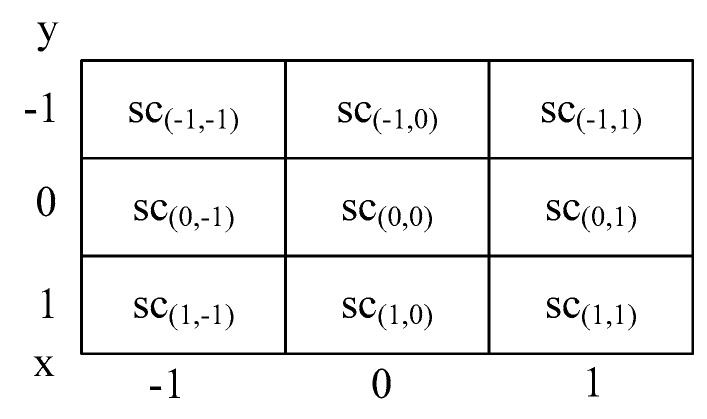
A FAST-BRISK 3×3 score patch.

In order to better determine the location of interest point, we use three 3×3 score patches which include the FAST scores of the interest point and 26 pixels surrounding it in the scale pyramid. The three 3×3 score patches are described by three levels, the first patch at the octave level below the interest point, the second patch at the level of the interest point, and the third patch at the level above the interest point. In the patch, the value at each pixel is equal to that pixel’s FAST score, which is denoted by $sc_{\left(i,j\right)}$, where i and j signify the position of the scored pixel relative to the interest point in space. For example, if the interest point is located at (1,1,0), the score $sc_{\left(-1,0\right)}$ will be located at (0,1,0). Figure 4 shows an instance of score patch. The FAST score maxima of three octave levels are denote by $sc\prime_{\left(-1\right)}$, $sc\prime_{\left(0\right)}$, and $sc\prime_{\left(1\right)}$, where a subscript of −1 and a subscript of 1 represent respectively the level below and above the interest point. The positions of these three maximum score are denoted by $\Delta x\prime_{\left(-1\right)}$, $\Delta x\prime_{\left(0\right)}$ and $\Delta x\prime_{\left(1\right)}$, where $\Delta x\prime_{\left(0\right)}=(\Delta x\prime_{\left(0\right)},\Delta y\prime_{\left(0\right)})$.

For solving the maximum sc′ and its position x′ of three score patches, we fit the parameters of the 2-D quadratic function given in Equation (12) to obtain the location and score of the quadratic’s local maximum.

(12) $$sc=ai^{2}+bij+cj^{2}+di+ej+f$$

The parameter φ of the 2-D quadratic function is solved by using the least square fit. The 2-D quadratic function is represented by the matrix form as:

(13) $$sc=\varphi^{T}w(i,j)$$

where $\varphi ={\left[a,b,c,d,e,f\right]}^{T}$, $w(i,j)={\left[i^{2},ij,j^{2},i,j,1\right]}^{T}$.

The least square fit can be signified as the minimization of the energy function E(φ) shown in Equation (14):

(14) $$E(\varphi )=\sum_{i=-1}^{1}\sum_{j=-1}^{1}{\left(\varphi^{T}w(i,j)-sc_{i,j}\right)}^{2}$$

Setting the derivative for φ with Equation (14) equal to zero and solving for φ yields Equation (15):

(15) $$\varphi =P^{-1}Wsc$$

where $P=\sum_{i=-1}^{1}\sum_{j=-1}^{1}w(i,j)w{\left(i,j\right)}^{T}$, W=[w(−1,−1) w(0,−1) ⋯ w(1,1)], $sc={\left[sc_{\left(-1,-1\right)},sc_{\left(0,-1\right)},sc_{\left(1,-1\right)},\cdots sc_{\left(1,1\right)}\right]}^{T}$.

Assuming the determinant of Hessian H(sc) passes the second derivative test stated in Equation (16):

(16) $$\begin{array}{l} H(sc)=\left[\begin{array}{l} \frac{\partial^{2}sc}{\partial i^{2}}\;\frac{\partial^{2}sc}{\partial i\partial j}\\ \frac{\partial^{2}sc}{\partial i\partial j}\;\frac{\partial^{2}sc}{\partial j^{2}} \end{array}\right]\\ \det(H(sc))=\frac{\partial^{2}sc}{\partial i^{2}}\frac{\partial^{2}sc}{\partial j^{2}}-\frac{\partial^{2}sc}{\partial i\partial j}\frac{\partial^{2}sc}{\partial j\partial i}<0 \end{array}$$

The local maximum for Equation (12) is found at the location Δx′ where the partial derivatives with respect to i and j are 0. The partial derivatives are stated in Equation (17):

(17) $$\begin{array}{l} \frac{\partial sc}{\partial i}=2ai+bj+d\\ \frac{\partial sc}{\partial j}=2cj+bi+e \end{array}$$

Setting the partial derivatives to 0 and solving for the location (Δx′,Δy′) represented by (i,j) in Equation (17), which is given in Equation (18).

(18) $$\begin{array}{l} \Delta x\prime ={\left(\Delta x\prime ,\Delta y\prime \right)}^{T}\\ \Delta x\prime =\frac{be-2cd}{4ac-b^{2}}\\ \Delta y\prime =\frac{bd-2ae}{4ac-b^{2}} \end{array}$$

The maximum score is then obtained by Equation (19):

(19) $$\begin{array}{cc} \mathrm{sc}\prime & =\varphi^{T}w(\Delta x\prime ,\Delta y\prime )\\ & =a{\left(\Delta x\prime \right)}^{2}+b(\Delta x\prime )(\Delta y\prime )+c{\left(\Delta y\prime \right)}^{2}+d(\Delta x\prime )+e(\Delta y\prime )+f \end{array}$$

### 4.3. Compute Scale Factor Using Depth Information

The BRISK algorithm detects feature points in the multi-scale pyramid model in order to ensure descriptors with scale invariance. This method is slow, and has a large memory requirement. By comparing the BRISK algorithm and other algorithms [36], we know that BRISK algorithm’s robustness of scale invariance is weak.

The FAST algorithm used to detect the feature points in this paper does not have a main direction. In order to make the descriptor have strong scale invariance robust, we will use the depth information of RGB-D image to compute scale factor [35]. Scale factor s of formula:

(20) $$s=\max(0.2,\;\frac{3.8-0.4\max(2,d)}{3})$$

where d is the depth of pixel point, max(2,d) denotes the filtering pixels with depth smaller than 2 meter.

### 4.4. Orientation by Intensity Centroid

The local orientation of interest in the BRISK algorithm is oriented by long-distance point pair. According to [36], it shows that the robust of rotation invariance is weak.

Feature points detected by the FAST algorithm do not have a main local direction, in order to make the descriptor with strong robust rotational invariance, we will use the Intensity Centroid [34] to orient the main direction of the feature point. The intensity centroid assumes that a corner’s intensity is offset from its center, and this vector may be used to impute an orientation. Rosin defines the moments of a patch as:

(21) $$m_{pq}=\sum_{x,y}x^{p}y^{q}I(x,y)$$

where x and y are relative to the position of the feature point, x,y∈[−r,r], r is the radius of neighborhood of FAST interest point, the values of q, p are “1” or “0”, I(x,y) is the gray intensity of point (x,y).

So, the centroid can be defined by $m_{00}$, $m_{01}$ and $m_{10}$:

(22) $$C=(\frac{m_{10}}{m_{00}},\frac{m_{01}}{m_{00}})$$

Corner center O to the centroid vector C is $\vec{OC}$, the orientation of local neighborhood of interest point is:

(23) $$\theta =\arctan(\frac{m_{01}}{m_{10}})=\arctan(\frac{\sum_{x,y}yI(x,y)}{\sum_{x,y}xI(x,y)})$$

## 5. Experimental Results and Analysis

All the experiments are done using the same notebook computer, which has an Intel Core i7-4700HQ CPU and 8.0 GB RAM with the Ubuntu 14.04 64bit operation system. The images used in the experiment are taken by Kinect Xbox 360 in the lab, and the size of depth image and RGB image is 640 × 480 pixels. In the experiment, the SURF algorithm, BRISK algorithm and the BRISK_D algorithm are used to extract feature points and image registration. We have made comparative experiments on indoor images in our lab and Freiburg dataset (https://vision.in.tum.de/data/datasets/rgbd-dataset).

### 5.1. Indoor Images in Our Lab

**Figure 5:**
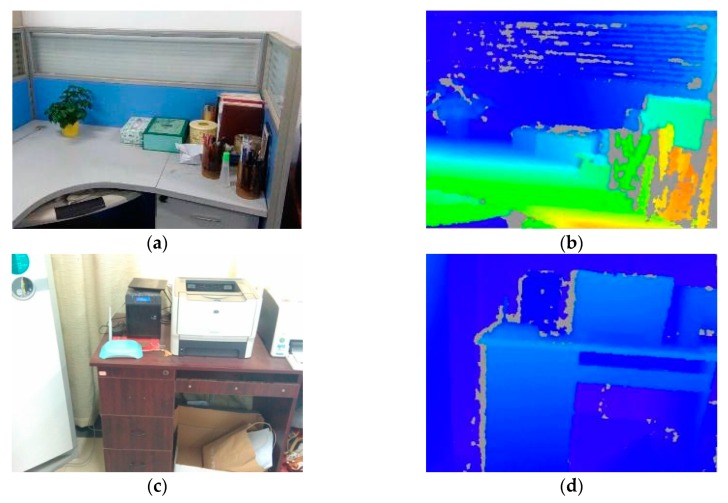
Reference images: (**a**) The RGB-D image of A; (**b**) The depth image of A; (**c**) The RGB-D image of B; (**d**) The depth image of B.

We have made comparative experiments on the lab objects, such as desk, chair, cup, corner debris. We select Figure 5a,c to make a detailed comparative analysis. Figure 5a,b are the RGB image of the reference Image A and the corresponding depth image. Figure 5c,d are the RGB image of the reference Image B and the corresponding depth image. The reference images A and B are in good light.

**Figure 6:**
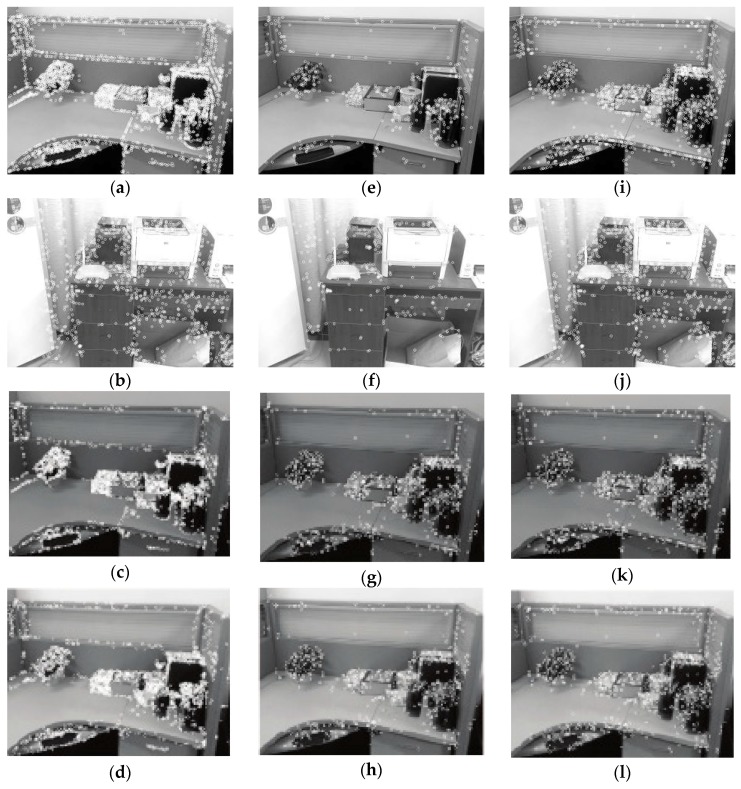
Feature points extracted by each algorithm. (**a**–**d**) Feature point detection using the Speeded Up Robust Feature (SURF) algorithm; (**e**–**h**) Feature point detection using the BRISK algorithm; (**i**–**l**) Feature point detection using BRISK_D algorithm. The first row (**a**,**e**,**i**) represents the comparison of the three algorithms with Image A; The second row (**b**,**f**,**j**) represents the comparison of the three algorithms with Image B; The third row (**c**,**g**,**k**) represents the comparison of the three algorithms with Image C.; The fourth row (**d**,**h**,**l**) represents the comparison of the three algorithms with Image D.

**Figure 7:**
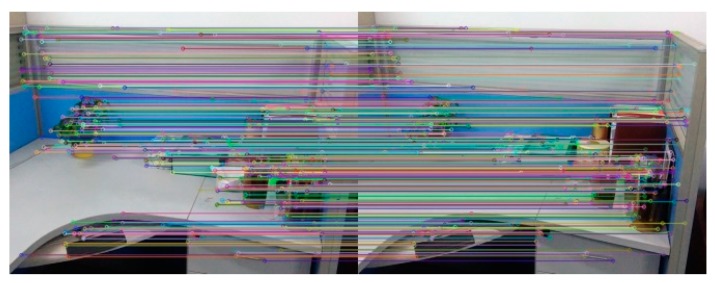
Experimental results of Image A registering with its own.

**Figure 8:**
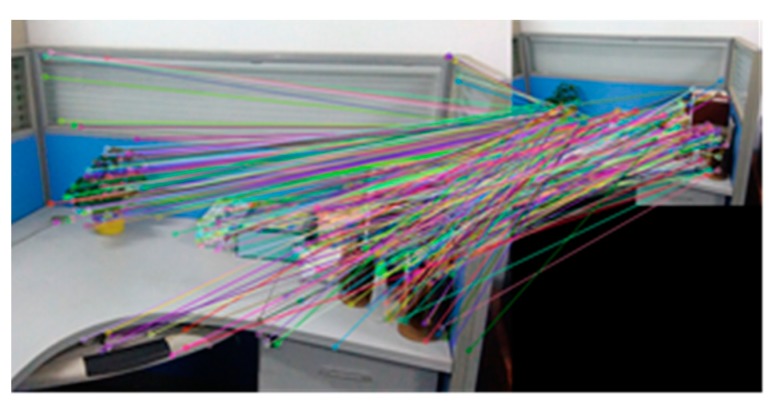
The experimental result about registering Image A with its own reducing to 1/2.

**Figure 9:**
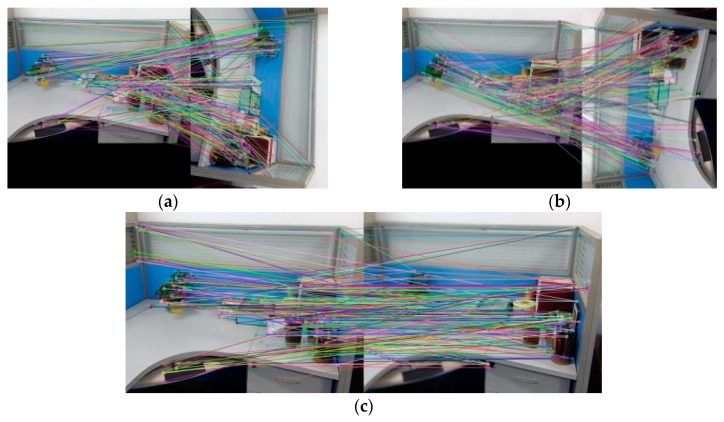
The registration after rotating reference image: (**a**) Rotating 90°; (**b**) Rotating 270°; (**c**) Rotating 180°.

**Figure 10:**
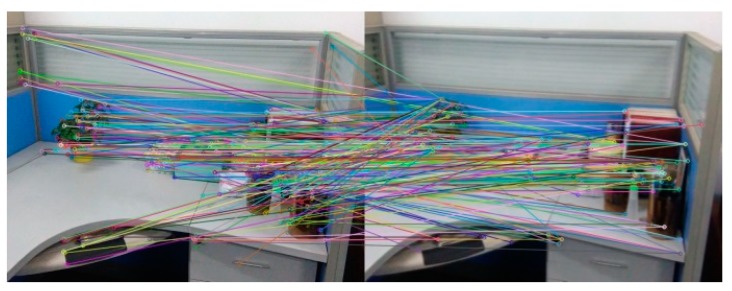
Image A registered with its own after blurring.

**Figure 11:**
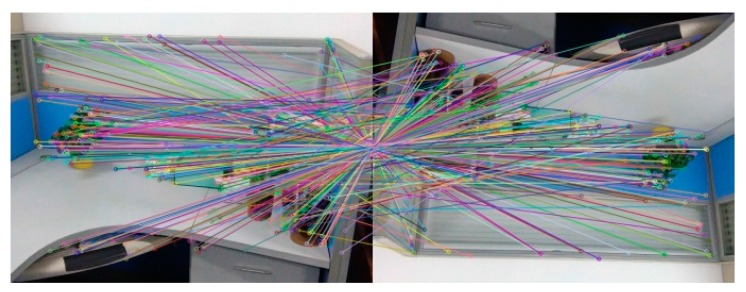
Image A registered with its own after local blur processing.

In the experiment, the SURF algorithm, the BRISK algorithm and the BRISK_D algorithm are used to extract and register the feature points for the collected reference images and the processed reference images respectively. The experimental results are shown in Table 1, Table 2, Table 3, Table 4, Table 5 and Table 6, Figure 6, Figure 7, Figure 8, Figure 9, Figure 10 and Figure 11.

Table 1 and Figure 6 show the performance test results of the algorithms for feature extraction. Column 1~3 in Figure 6 are experimental results of feature point extraction for images by the SURF algorithm, the BRISK algorithm and the BRISK_D algorithm. The image C in Table 1 is the image that Image A reduces brightness, Image D is the result of Image A adding the Gaussian Blur. The depth images of C and D are the same as those of A. Table 1 shows the number of feature points and the time required to extract the feature from Image A to D using above three algorithms. From Figure 6 and Table 1, the feature points extracted by the SURF algorithm are the most among the 3 different images. They are stable. The influence of uneven illumination and brightness change is small, but the time consumption is much longer than the other two algorithms, about 10 times that of the BRISK algorithm, and 9 times that of the BRISK_D algorithm. The BRISK algorithm has the least number of feature points, and is greatly affected by uneven illumination and brightness changes, but it has short time consumption. The number of feature point extracted by the BRISK_D algorithm is less than SURF algorithm and one time more than the BRISK algorithm. The influence of uneven illumination and brightness change is small, the time consumed is equivalent to the BRISK algorithm, but the distribution of feature points in this algorithm is more balanced than that of the other two algorithms.

Figure 7 and Table 2 are the experimental results about the efficiency of feature matching in the algorithm. Figure 7 is the experimental results that Image A registers with its own using the algorithm in this paper. Table 2 represents the correct matching number of feature points for Image A and Image B registering with its own and the time required for feature matching using the SURF algorithm, the BRISK algorithm and the BRISK_D algorithm respectively. It can be seen from Table 2 that the correct matching number of feature points of the SURF algorithm is the most, almost five times more than the BRISK algorithm and two times more than the BRISK_D algorithm, and the time consumption of the SURF algorithm is almost ten times more than the BRISK algorithm and five times more than the BRISK_D algorithm. Considering that correct matching number of feature points of the BRISK_D algorithm is approximately two times that of the BRISK algorithm, the matching speed of BRISK algorithm is similar to that of this algorithm, but much faster than that of SURF algorithm.

Figure 8 and Table 3 are the testing experimental results of scale invariance of algorithms. Figure 8 is the experimental result that Image A registers with the image reducing A to 1/2 (Image A × 0.5) using the BRISK_D algorithm. Table 3 represents the correct matching number of feature points that Image A and Image B register with their own after scaling respectively using the SURF algorithm, the BRISK algorithm and the BRISK_D algorithm. It can be shown from Table 3: the correct feature points of the SURF algorithm are the most, and they are less affected by scale changes of the image. The correct matching number of feature points of the BRISK algorithm are the least, and they are easily affected by scale changes of image. Compared with the BRISK algorithm, the BRISK_D algorithm is less affected by scale changes and the correct matching number increases 87% at least. However, when the image magnification is 4 times, the correct number of matching drops obviously.

The Figure 9 and the Table 4 are experimental results which test rotation invariance of the algorithm. Figure 8a–c are the experimental results of registration between Image A and Image A after rotating 90°, 270° and 180° using BRISK_D algorithm. Table 4 represents the correct matching number of feature points using the SURF algorithm, the BRISK algorithm and the BRISK_D algorithm respectively to register reference image and it after rotating with their own. It can be seen in Table 4 that after rotating, the SURF algorithm has more correct matching number of feature points; the correct matching number of the BRISK algorithm is the least; the correct matching number of the BRISK_D algorithm is less than SURF algorithm but is 71% more than the BRISK algorithm, and its stability is better than BRISK algorithm.

The Figure 10, Figure 11 and the Table 5 are experimental results that algorithms test blur invariance of the image. In Table 5, the images A1 to A5 are respectively blurred for Image A with Gaussian kernel as 0.6, 1.1, 1.6, 2.1 and 2.6. The Image A6 and Image A7 are respectively blurred for left half and right half of Image A with Gaussian kernel as 0.6. Table 5 represents the correct matching number of feature points that the Image A registers with its own after blurring respectively using the SURF algorithm, the BRISK algorithm and the BRISK_D algorithm. Figure 10 is the experimental result that the Image A registers with Image A1 using the algorithm in this paper, and Figure 11 is the experimental result that the Image A registers with Image A6 using the algorithm in this paper. It can be shown from the experimental results of images A1 to A5 in Table 5 that the correct matching number of feature points of SURF algorithm under different Gaussian Blur is the most. With the increase of the Gaussian kernel, the number of correctly matched feature points decreases, but the number of correctly matched feature points is still large. The correct matching number of BRISK algorithm and the BRISK_D algorithm decreases significantly when the degree of blur is little (Gaussian kernel is 0.6 or 1.1), but with the degree of blur increases, the correct matching number is stable after Gaussian kernel coming to 1.6. It can be seen from the experimental results of Image A6 and A7: when the image is locally blurred, the correct matching number of feature points of the SURF algorithm is the most, and its performance is most stable; the correct matching number of feature points of the BRISK algorithm is the least and it is greatly influenced by blur. The correct matching number of feature points of the BRISK_D algorithm is less than SURF algorithm, but is at least 35% more than BRISK algorithm, and its stability is higher than BRISK algorithm.

Table 6 is the experimental result that tests the invariance of illumination variations of the algorithms. Image B1 to B5 respectively represent the images that their brightness reduces 10%, 20%, 30%, 40% and 50%. Image B6 and B7 respectively represent the images that the upper half and lower half of Image A reduce its brightness to 50%. It can be shown from the experimental result of Image B1 to B5 in Table 6 that when the intensity of the illumination is changed, the correct matching number of feature points of the SURF algorithm is the most and its range of change is the least; the correct matching number of feature points of BRISK algorithm is the least and its range of change is the largest; the correct matching number of feature points of the algorithm in this paper is less than SURF algorithm but 27% more than BRISK algorithm at least, and its range of quantity change is also less than BRISK algorithm. It can be shown from the experimental result of Image B6 and B7 in Table 6 that when the distribution of illumination is uneven, the correct matching number of feature points of SURF algorithm is the most, and it has the least influence; the correct matching number of feature points of BRISK algorithm is the least, and it has the most influence; the correct matching number of feature points of the BRISK_D algorithm is less than SURF algorithm but 13% more than BRISK algorithm at least.

### 5.2. Freiburg Dataset

**Figure 12:**
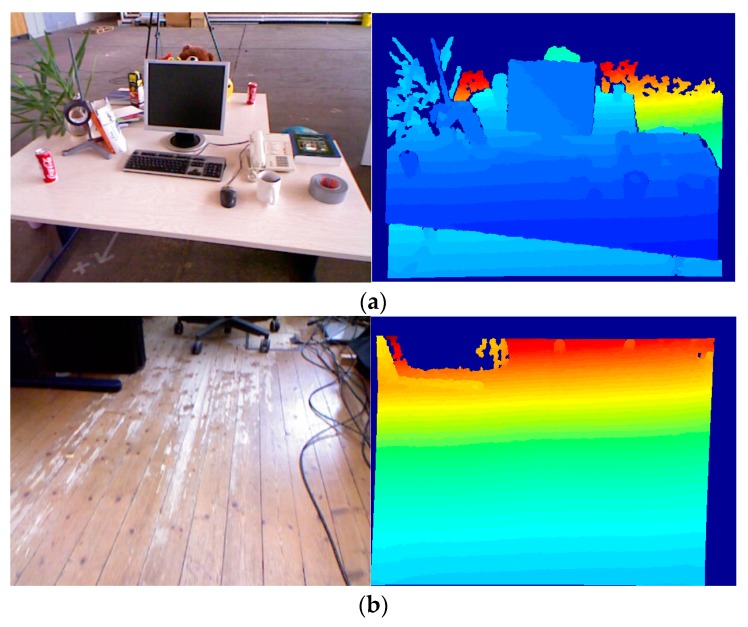

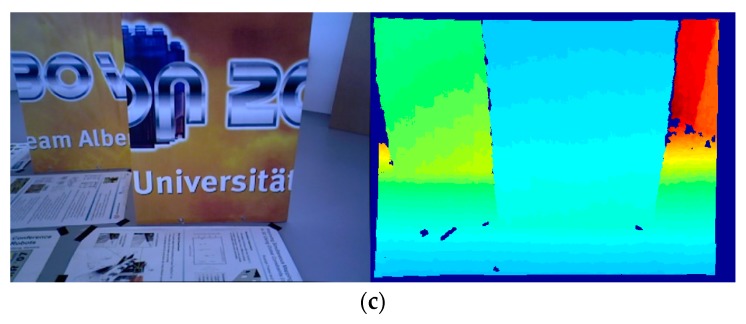
Images from three sequences of Freiburg dataset. (**a**) RGB image and depth image of Desk sequence (**b**) RGB image and depth image of Floor sequence (**c**) RGB image and depth image of structure texture far sequence.

Freiburg dataset consists of several indoor RGBD image sequences of 640 × 480 pixels acquired with Microsoft Kinect and ASUS Xtion sensors. This dataset is suitable for SLAM and visual odometry experiments. We use three sequenced of RGBD images Freiburg dataset, containing more complex camera position changes: sequences desk (42 frames with 10 frames skipping), structure texture far (61 frames with 5 frames skipping) and floor (21 frames with 5 frames skipping). Several images from three sequences are shown in Figure 12. The depth maps are of a standard Kinect quality.

**Figure 13:**
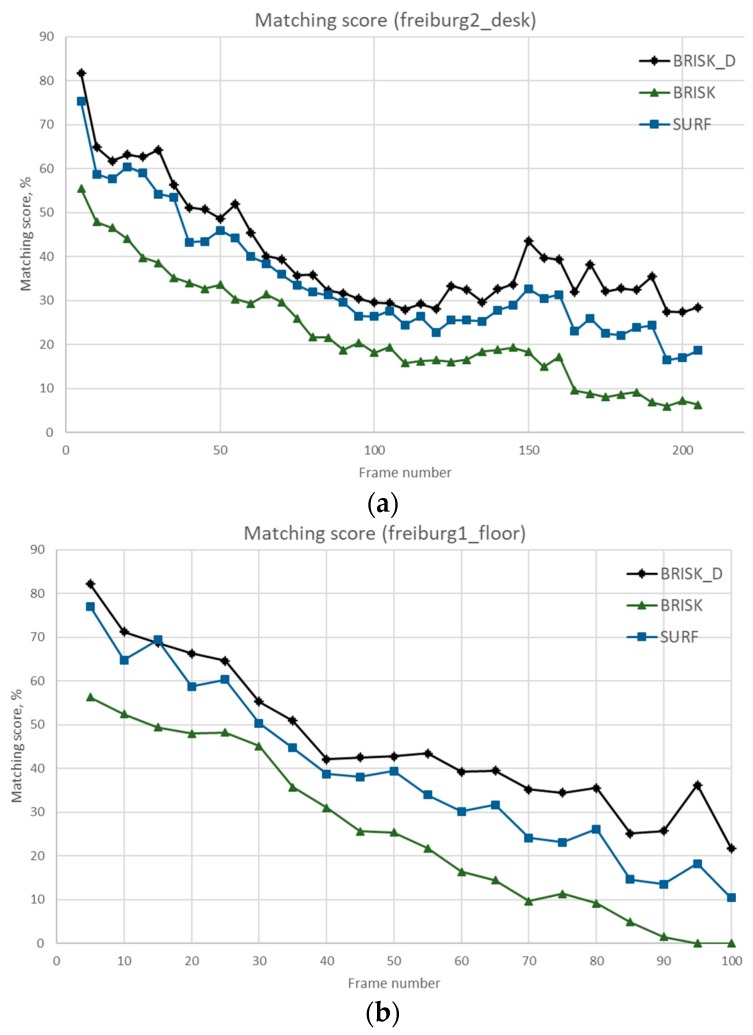

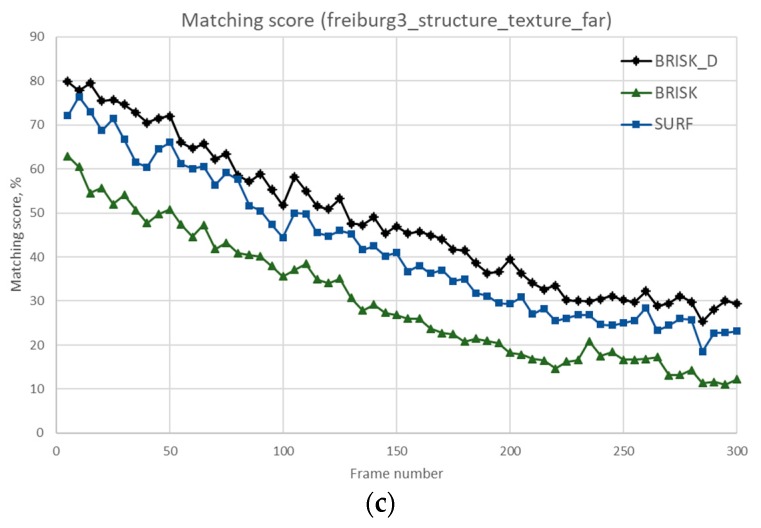
Comparison of three different algorithms on three sequences (**a**) Comparison of Matching score on Desk sequence (**b**) Comparison of Matching score on Floor sequence (**c**) Comparison of Matching score on structure texture far sequence.

In each sequence, its first image is taken as the reference, the k-th image (k=2,3,⋯⋯) of the sequence is then matched against this reference. The matching process is as follows. A set of local features extracted from the first image is matched against the feature set from the k-th image. Let $F_{k}$ denote the set of features found in the k-th image. We compare the three algorithms by calculating the Matching scores (shown in Figure 13). Matching score computation: the ratio between the number of correct matches and the maximum possible number of matches is reported as matching score per image pair. For k-th image, the formula [37] of the Matching score is as follow:

(24) $$M_{\varepsilon_{0}}(k)=\frac{N_{\varepsilon_{0}}(F_{1},F_{k})}{\min(|F_{1}|,|F_{k}|)}$$

The Matching score shows how many features in percentage are actually repeatable in each test image with respect to the reference image. This measures the performance of the detector.

From the comparison results of Figure 13, we can see that the overall performance of the Matching score is on a downward trend. Freiburg dataset is recorded data by holding the Kinect camera and moving slowly during recording. When recording data, the camera is constantly panned and rotated, and the camera pose changes constantly. So, it’s normal for Matching score to show a downward trend. From the comparison results, the BRISK_D algorithm has the best Matching score, SURF algorithm is the second, and BRISK is the worst. The BRISK_D algorithm combines depth information and has certain advantages for matching video images in the room.

## 6. Conclusions

This paper improves the BRISK algorithm using the depth information of the RGB-D image, and computes scale factors in order to increase the scale invariance of descriptor, then uses Intensity Centroid to determine the main direction of feature points in order to increase the scale invariance. Experimental results show that the algorithm in this paper is fast and its scale invariance and rotational invariance are stronger than the original BRISK algorithm; the algorithm can also achieve better matching results when the illumination is changed and image is blurred. But when the image has a large-scale change, the accuracy of the algorithm in this paper decreases significantly, and when the image is blurred, the stability of this algorithm is not stronger. The next step in this work is to focus on enhancing the stability of this algorithm under the condition that image has a large-scale change and image is blurred.

## Figures and Tables

**Table 1:** The comparison about feature points extracted by each algorithm and their time consumption.

Reference Image	SURF Algorithm		BRISK Algorithm		BRISK_D Algorithm	
	Quantity	Time (/ms)	Quantity	Time (/ms)	Quantity	Time (/ms)
Image A	2065	466	469	47	1099	51
Image B	1467	301	323	31	726	32
Image C	1945	374	384	38	821	39
Image D	1863	324	210	32	678	35

**Table 2:** The comparison about correct matching number of feature points and time consumption (/ms) by each algorithm.

Reference Image	SURF Algorithm		BRISK Algorithm		BRISK_D Algorithm	
	Quantity	Time (/ms)	Quantity	Time (/ms)	Quantity	Time (/ms)
Image A	1941	186	443	17	1045	41
Image B	1356	113	318	12	719	28

**Table 3:** The correct matching number of reference image after scaling.

Reference Image	Scaling Bits	SURF Algorithm	BRISK Algorithm	BRISK_D Algorithm
Image A	0.25	1052	67	213
	0.5	1299	105	258
	2	1145	91	289
	4	985	84	157
Image B	0.25	689	39	105
	0.5	744	59	142
	2	763	61	127
	4	587	32	118

**Table 4:** The correct matching number of reference image after rotating.

Reference Image	Rotation Angle	SURF Algorithm	BRISK Algorithm	BRISK_D Algorithm
Image A	0	1941	443	1045
	90	774	124	316
	180	1052	162	388
	270	875	107	294
Image B	0	1398	284	689
	90	561	97	197
	180	812	124	215
	270	498	84	156

**Table 5:** The correct matching number of 3 algorithms after image blurring.

Reference Image	SURF Algorithm	BRISK Algorithm	BRISK_D Algorithm
Image A1	1698	257	645
Image A2	1427	127	317
Image A3	1124	71	164
Image A4	964	65	147
Image A5	681	57	138
Image A6	1823	327	467
Image A7	1532	294	398

**Table 6:** The correct matching number of 3 algorithms after reducing the image brightness.

Reference Image	SURF Algorithm	BRISK Algorithm	BRISK_D Algorithm
Image B1	1857	375	517
Image B2	1654	294	452
Image B3	1566	212	374
Image B4	1521	175	315
Image B5	1320	154	286
Image B6	1473	261	321
Image B7	1527	314	356
